# Perception of unrelated microbe-associated molecular patterns triggers conserved yet variable physiological and transcriptional changes in *Brassica rapa* ssp. *pekinensis*

**DOI:** 10.1038/s41438-020-00410-0

**Published:** 2020-11-01

**Authors:** Wanhui Kim, Maxim Prokchorchik, Yonghua Tian, Seulgi Kim, Hyelim Jeon, Cécile Segonzac

**Affiliations:** 1 Department of Plant Science, Plant Genomics and Breeding Institute and Research Institute of Agriculture and Life Sciences, Seoul National University, Seoul, 08826 Republic of Korea; 2 Plant Immunity Research Center, Seoul National University, Seoul, 08826 Republic of Korea; 3 Life Sciences Department, Pohang University of Science and Technology, Pohang, 37673 Republic of Korea; 4 Department of Agriculture, Forestry and Bioresources, Seoul National University, Seoul, 08826 Republic of Korea

**Keywords:** Pattern recognition receptors in plants, Biotic

## Abstract

Pattern-triggered immunity (PTI) includes the different transcriptional and physiological responses that enable plants to ward off microbial invasion. Surface-localized pattern-recognition receptors (PRRs) recognize conserved microbe-associated molecular patterns (MAMPs) and initiate a branched signaling cascade that culminate in an effective restriction of pathogen growth. In the model species *Arabidopsis thaliana*, early PTI events triggered by different PRRs are broadly conserved although their nature or intensity is dependent on the origin and features of the detected MAMP. In order to provide a functional basis for disease resistance in leafy vegetable crops, we surveyed the conservation of PTI events in *Brassica rapa* ssp. *pekinensis*. We identified the PRR homologs present in *B. rapa* genome and found that only one of the two copies of the bacterial Elongation factor-Tu receptor (EFR) might function. We also characterized the extent and unexpected specificity of the transcriptional changes occurring when *B. rapa* seedlings are treated with two unrelated MAMPs, the bacterial flagellin flg22 peptide and the fungal cell wall component chitin. Finally, using a MAMP-induced protection assay, we could show that bacterial and fungal MAMPs elicit a robust immunity in *B. rapa*, despite significant differences in the kinetic and amplitude of the early signaling events. Our data support the relevance of PTI for crop protection and highlight specific functional target for disease resistance breeding in Brassica crops.

## Introduction

Perception of non-self or damaged-self is essential for plants to adjust to environmental changes and is of particular importance to ward off pathogen invasion. Plants have evolved two major sets of receptors that recognize the presence of microbes and activate defense mechanisms that efficiently restrict pathogen growth^1^. Microbe-associated molecular patterns (MAMPs) are conserved features essential for microbial survival. Characterized MAMPs include components of the bacterial or fungal cell membrane and cell wall (lipopolysaccharides, peptidoglycan, chitin), the building block of the bacterial flagellum (flagellin) or even components of the general cell machinery (bacterial elongation factor-Tu)^2^. At the plant-microbe interface, MAMPs may bind to the extracellular domain of the plant pattern-recognition receptors (PRRs), leading to activation of conserved sets of defense responses including a wide transcriptional reprograming, hormonal changes, reinforcement of the plant cell wall and production of antimicrobial compounds^3^. Plants lacking PRRs are generally more susceptible to pathogens, highlighting the contribution of the pattern-triggered immunity (PTI) to plant disease resistance^4^.

Multiple PRRs have been genetically identified and characterized in the model species *Arabidopsis thaliana* and few other species such as tomato or rice^5^. PRRs are plasma membrane-localized proteins predominantly belonging to the receptor-like kinase or the receptor-like protein families^5^. Some PRRs such as FLAGELLIN SENSING 2 (FLS2) or CHITIN ELICITOR RECEPTOR KINASE 1 (CERK1) are broadly conserved across the plant kingdom, while others such as ELONGATION FACTOR TU RECEPTOR (EFR) or LIPOPOLYSACCHARIDE-SPECIFIC REDUCED ELICITATION (LORE) are only present in the *Brassicaceae* family^6,7^. Following MAMP binding, the activation mechanisms of PRRs are diverse and often include the recruitment of other membrane-associated proteins, as co-receptors or as additional signaling components^8^. PRR activation triggers two branches of signaling events, one leading to a characteristic influx of Ca^2+^ and the production of reactive oxygen species (ROS) in the apoplast; the other mediated by several classes of kinases that leads to a significant transcriptional reprogramming of the cell^9^. These early PTI events are broadly conserved among distant plant species, therefore the transfer of PRR is sufficient to confer novel MAMP detection capacity to unrelated plant families^10^. *Brassica rapa* ssp. *pekinensis* is an important vegetable crop in North-East Asia, closely related to the model species Arabidopsis. A chromosome-level assembly of *B. rapa* genome is available and has recently been surveyed for components of disease resistance^11,12^. Although a broad conservation with the well-characterized genetic components of PTI in Arabidopsis could be inferred from the close relationship between the two species, the MAMP detection system and PTI signaling events in *B. rapa* remain to be characterized. In the aim to provide relevant targets to improve disease resistance in Brassica crops, we have tested the functionality of the two copies of the EFR homologs present in *B. rapa* genome and characterized further the physiological and transcriptional responses of *B. rapa* to several unrelated MAMPs.

## Results

### Identification and expression of pattern-recognition receptor gene homologs in *B. rapa* genome

Prior to test the range of MAMPs detected by *B. rapa*, we surveyed its genome for the presence of pattern-recognition receptor (PRR) gene homologs (Table 1, Fig. S1). Using the search for syntenic ortholog tool developed by Cheng et al.^13^, we identified two putative homologs of *AtFLS2, AtEFR, LYSINE-MOTIF DOMAIN PROTEIN 1 (AtLYM1)* and *LYSINE-MOTIF DOMAIN PROTEIN 2 (AtLYM2)*, mostly located in the moderate gene fractionation portion of the genome^7,14–17^. We could identify only one syntenic ortholog of *AtCERK1* and *LysM-CONTAINING RECEPTOR-LIKE KINASE 5* (*AtLYK5)*^15,18^. No syntenic orthologs could be found for *LYSINE-MOTIF DOMAIN PROTEIN 3* (*AtLYM3)* and *AtLORE* required, respectively, for the perception of bacterial peptidoglycan and lipopolysaccharides^6,17^. However, *Bra031434* and *Bra008320* showed the highest amino acid identity with *AtLORE* (93.1%) and *AtLYM3* (91.3%) and were considered as non-syntenic homologs for these receptors.

**Table 1:** Identification and expression of pattern-recognition receptor homologs in *B. rapa* genome

Pattern	Receptor	*A. thaliana* gene ID	*B. rapa* gene ID (subgenome)^a^	Expression (FPKM)^b^		
				Root	Stem	Leaf
Chitin	CERK1	AT3G21630	Bra031293 (LF)	8.20	4.43	4.55
	LYK5	AT2G33580	Bra021861 (MF1)	61.03	68.10	37.97
	LYM2	AT2G17120	Bra002021 (LF)	21.93	34.51	22.89
			Bra009660 (MF2)	14.98	26.23	13.47
EF-Tu (elf18)	EFR	AT5G20480	Bra002305 (LF)	1.29	0.38	0.99
			Bra006560 (MF1)	0.22	0.11	0.20
Flagellin (flg22)	FLS2	AT5G46330	Bra022032 (MF1)	0.00	0.00	0.00
			Bra017563 (MF2)	4.59	4.51	5.97
Lipopolysaccharides	LORE	AT1G61380	Bra031434 (ns)	0.00	1.62	10.33
Peptidoglycan	LYM1	AT1G21880	Bra017956 (LF)	26.01	50.49	11.57
			Bra016402 (MF1)	40.64	92.87	33.98
	LYM3	AT1G77630	Bra008320 (ns)	27.29	52.13	7.93

*LF* lest gene fractionation, *MF1* moderate gene fractionation, *MF2* most gene fractionation, *ns* non-syntenic.

^a^From Cheng et al.^13^.

^b^From Tong et al.^19^.

Using available RNA-seq data from *B. rapa* tissues^19^, we have collected expression values for each of these putative PRR encoding genes in root, stem, and leaf (Table 1). In these three tissues, the genes coding for polysaccharide chitin and peptidoglycan receptors were the most abundantly expressed. *LORE* non-syntenic homolog *Bra031434* expression was not detected in roots but reached a similar expression level as *LYM1* and *LYM2* homologs in leaf tissues. The next most expressed genes were the ones coding for the proteinaceous flagellin and Elongation factor-Tu receptors, *Bra017563* (hereafter *BraFLS2*) and *Bra002305* (hereafter *BraEFR2*). Interestingly, the transcript of the other putative copy of *FLS2* (*Bra022032*) was not detected in this study whereas the second putative copy of *EFR* (*Bra006560*, hereafter *BraEFR1*) was, albeit to a lower level than *BraEFR2*. Altogether, these data hinted at the presence and expression of PRRs that could perceive bacterial (EF-Tu, flagellin, lipopolysaccharides, peptidoglycan) and fungal (chitin) patterns in *B. rapa* vegetative tissues.

### Only one of the two *B. rapa* EFR homologs is functional

EFR is the *Brassicaceae*-specific receptor for the bacterial Elongation Factor-Tu^7,20^. Unlike *BraFLS2*, with only one of the two copies expressed, both copies of *BraEFR* are expressed in vegetative tissues (Table 1). Protein sequence alignment with AtEFR revealed higher level of identity (78.2% vs 76.8%) and similarity (91.8% vs 91.3%) for BraEFR1 compared to BraEFR2 (Fig. S2). Both proteins harbor the conserved 21 leucine-rich repeat (LRR) domains of AtEFR and the ATP-binding and proton-acceptor sites in the kinase domain. To determine whether both copies of BraEFR are functional, we cloned *AtEFR*, *BraEFR1,* and *BraEFR2* coding sequences in C-terminal fusion with 6 hemagglutinin (HA) repeats or the yellow fluorescent protein (YFP) and under the control of the constitutive CaMV 35S promoter for Agrobacterium-mediated expression *in planta*.

**Fig. 1:**
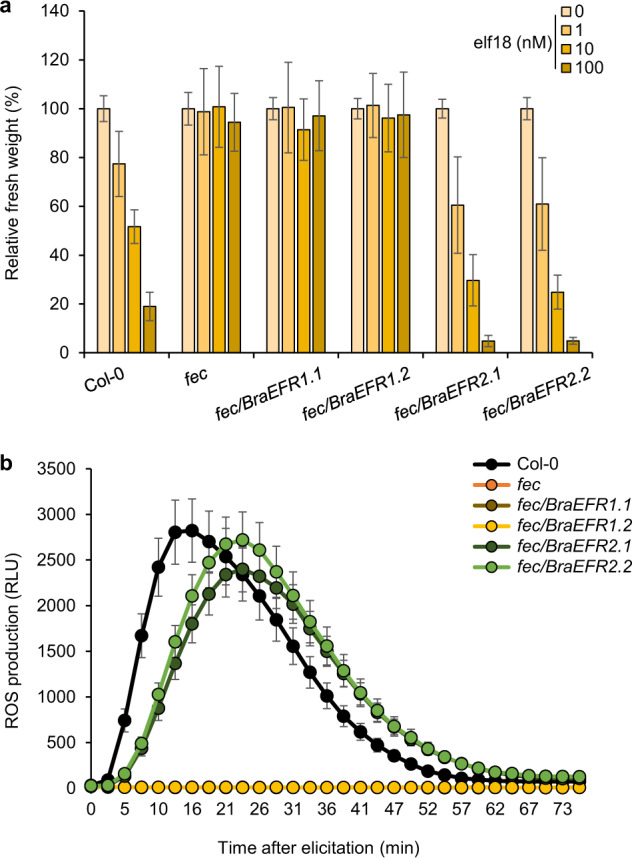
Only one of the two B. rapa EFR orthologs is functional. **a** Seedling growth inhibition in Arabidopsis wild type (Col-0), *fls2 efr cerk1* (*fec*) triple mutant and two independent *fec* transgenic lines expressing either *BraEFR1* (*fec/BraEFR1-1*, *fec/BraEFR1-2*) or *BraEFR2* (*fec/BraEFR2-1*, *fec/BraEFR2-2*). Seedlings were grown in medium supplemented with increasing concentration of elf18 and fresh weight determined after 12 days. Data are presented relative to seedling fresh weight in the medium without elf18 and are mean values +/− standard error from three independent biological repeats (*n* = 18). **b** Kinetic of elf18-elicited ROS production in Col-0, *fec*, *fec/BraEFR1-1*, *fec/BraEFR1-2*, *fec/BraEFR2-1* and *fec/BraEFR2-2*. Leaf discs from soil-grown 5-week-old plants were treated with 50 nM elf18 for 75 min. Data are mean values +/− standard error of relative light unit (RLU) from three independent biological repeats (*n* = 48)

We transformed Arabidopsis *fls2 efr cerk1* (*fec*) triple mutant that lacks a functional EFR and is therefore irresponsive to treatment with elf18 peptide to test BraEFR function^7,20,21^. We obtained independent *fec* transgenic lines expressing either *BraEFR1* or *BraEFR2* to a similar level as *EFR* in Col-0 plants (Fig. S3). Seedling growth inhibition in presence of defense-eliciting peptides such as flg22, elf18 or Atpep1 is a widely used method to rapidly assess peptide perception in Arabidopsis^7,16,22^. We could measure a dose-dependent reduction in fresh weight of Col-0 seedlings exposed to increasing concentration of elf18 (Fig. 1a). As expected, the fresh weight of *fec* seedlings was not affected by elf18 supplement in the growth medium. However, two independent *fec/BraEFR2* lines showed dose-dependent growth inhibition, whereas no change in fresh weight was detected in *fec/BraEFR1* lines (Fig. 1a). To examine whether *BraEFR* alleles could complement the *fec* mutant for earlier elf18-triggered event, we monitored the rapid production of reactive oxygen species (ROS) in the same lines (Fig. 1b). As expected, we could detect a fast and transient ROS production in Col-0 <2 min after elf18 elicitation but not in *fec* plants. The elf18-triggered ROS production was restored to wild type level in *fec/BraEFR2* lines but completely absent from the *fec/BraEFR1* lines (Fig. 1b).

We further assessed the gain of elf18 recognition in *Nicotiana benthamiana* that naturally lacks EFR^7^ by transient expression of AtEFR-, BraEFR1- or BraEFR2-YFP protein fusions. We measured the expression of the MAMP-responsive marker gene *NbCYP71D20*^23^, one hour after flg22 or elf18 treatment (Fig. S4a). *NbCYP71D20* expression was similarly induced in all the samples treated with flg22. Conversely, *NbCYP71D20* induction was only detected in leaf expressing *AtEFR* or *BraEFR2* after elf18 treatment. We could detect the fluorescence signal of both BraEFR1-YFP and BraEFR2-YFP at the periphery of *N. benthamiana* epidermal cells (Fig. S4b).

Altogether, these data indicate that although both alleles of *BraEFR* are expressed and no major difference with AtEFR in terms of protein sequence could be detected, only one isoform of BraEFR is functional *in planta*.

### Characterization of early responses to unrelated MAMPs in *B. rapa*

**Fig. 2:**
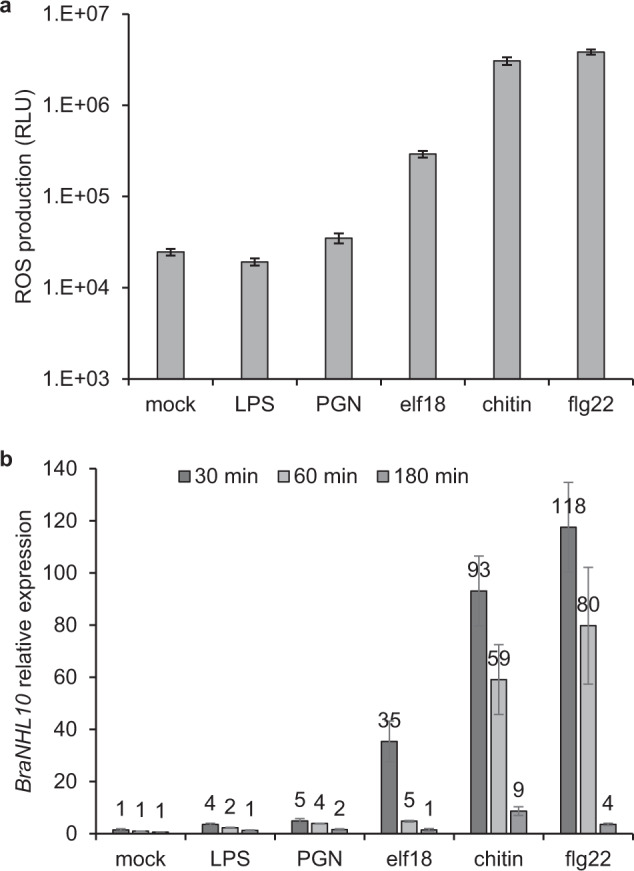
Different MAMPs elicit ROS burst and modulate gene expression in *B. rapa*. Norang leaf discs were treated with water (mock), 50 μg ml^−1^ LPS, 50 μg ml^−1^ PGN, 100 nM elf18, 100 μg ml^−1^ chitin or 100nM flg22. **a** Total ROS production during 75 min measurement. Data are mean values +/− standard error of relative light unit (RLU) from three independent biological repeats (*n* = 48). Note logarithmic scale. **b** *BraNHL10* (*Bra017272*) expression normalized by *BraEF1α* (*Bra006661*) and relative to 60 min mock-treated sample. Data are mean values +/− standard error from three independent biological repeats

To survey the range of MAMPs that can be detected in *B. rapa*, we measured ROS production and PTI-associated marker gene expression in the leaves of Norang, a commercial cultivar of *B. rapa*, upon treatment with bacterial and fungal MAMPs (Fig. 2). A significant ROS accumulation was detected within 5 min in Norang leaves treated with elf18, chitin, and flg22 compared to mock-, LPS- or PGN-treated leaves (Fig. 2a). Of note, elf18-triggered ROS production was at least one order of magnitude lower than the response elicited by chitin or flg22. Together with our analysis of the *BraEFR* genes, this suggests that although *B. rapa* possesses a functional receptor for elf18, the amplitude of the elicited response is much weaker than the one triggered by flagellin or chitin perception. Characteristics of ROS production in function of MAMP concentration were further assessed for elf18, chitin and flg22 (Fig. S5). We observed a clear positive correlation between kinetic and amplitude of ROS production and increasing dose of MAMPs, supporting the presence and functionality of PRRs in *B. rapa* leaf tissues.

Considering that some MAMPs could not elicit detectable ROS production or could trigger slower responses^6,17^, we also tested the induction of PTI-associated marker gene expression in Norang at 30, 60, and 180 min after treatment with different MAMPs (Fig. 2b). We identified *Bra017272* (hereafter *BraNHL10*) as a syntenic homolog of *AtNHL10*, a well-characterized marker gene rapidly regulated by MAMP treatment in Arabidopsis^24^. *BraNHL10* expression was unchanged after 30, 60, and 180 min of mock treatment but significantly upregulated following MAMP elicitation. Interestingly, we noticed a strong gradation in the intensity of *BraNHL10* induction from 4- to 5-fold upon treatment with LPS or PGN to ~30-fold with elf18 and ~100-fold with chitin or flg22 (Fig. 2b). In all cases, *BraNHL10* expression was rapidly upregulated after 30 min and gradually decreasing with prolonged (60 and 180 min) treatment. These results indicate first that *B. rapa* gene expression can rapidly change upon MAMP detection, as observed in the model species *A. thaliana*. Second, LPS and PGN could induce a significant change in gene expression in *B. rapa* although both MAMPs could not elicit a detectable ROS production.

### Transcriptomic reprogramming elicited by flg22 and chitin in *B. rapa*

Having established that chitin and flg22 could elicit the most robust physiological responses in *B. rapa*, we chose these two MAMPs to characterize the early transcriptional response. Leaf discs from Norang seedlings were incubated with water, chitin or flg22 for 30 and 180 min. Three biological replicates per treatment and time point were subjected to RNA-Seq. RNA Illumina sequencing yielded on average 58 million reads per sample, of which more than 94% aligned to the *B. rapa* genome^12^ after quality filtering (Tables S1 and S2). Differentially expressed genes (DEGs) were selected from each treatment and time point according to the significance in fold change expression (*p* < 0.05) and a threshold level of at least twofold-change in comparison with the water-treated controls that were harvested at the same time (Table S3). To confirm the analysis was performed correctly, we tested the expression of randomly selected DEGs by quantitative RT-PCR in other sets of biological samples and observed a high correlation between the results of the two quantification methods (Fig. S6).

**Fig. 3:**
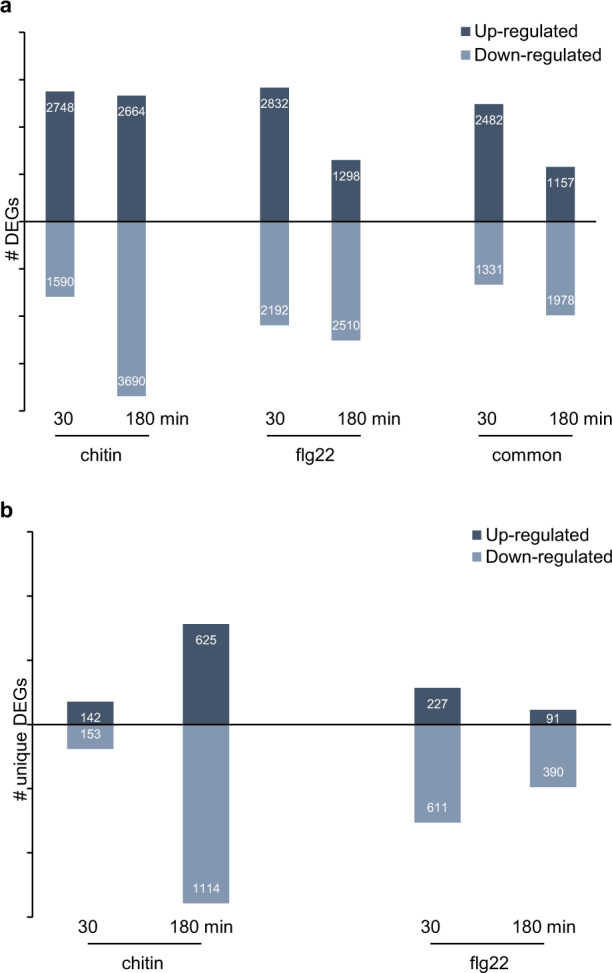
Overview of the transcriptional reprograming triggered by flg22 or chitin in *B. rapa*. Number of differentially expressed genes (DEGs; |fc| > 2 and *p* < 0.05) in chitin- and flg22-treated samples compared to corresponding water-treated samples after 30 and 180 min. **a** Total number of DEGs in each condition. Common represents DEGs detected in both chitin- and flg22-treated samples. **b** Number of DEGs uniquely detected in one condition (MAMP/time)

Both MAMPs elicited a substantial transcriptional reprogramming in Norang leaf, with a significant overlap at 30 min, comprising 2482 and 1331 genes commonly up- and downregulated respectively by flg22 and chitin (Fig. 3a). Interestingly, after 3 h of treatment, the extent of the transcriptional change was higher in chitin-treated leaves (2664 and 3690 up- and downregulated genes, respectively) compared to that in flg22-treated leaves (1298 and 2510 up- and downregulated genes, respectively). This prompted us to identify genes that were uniquely regulated in each condition. As shown in Fig. 3b, there are relatively few genes uniquely regulated by either MAMP at 30 min, with the exception of 611 downregulated genes in flg22-treated samples. Conversely, 625 up- and 1114 downregulated genes were uniquely detected in the leaves treated with chitin for 3 h.

**Fig. 4:**
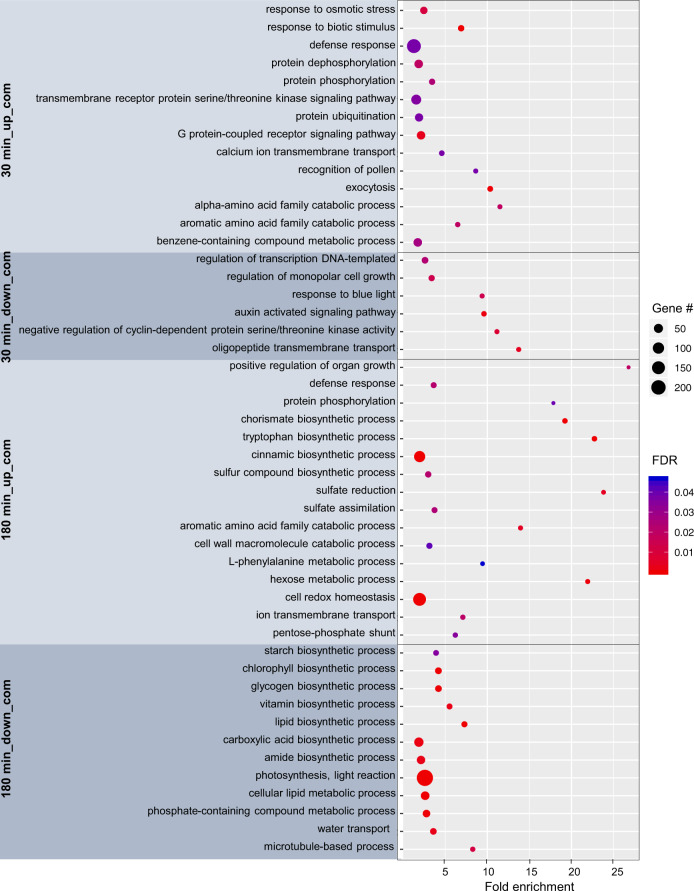
Biological functions over-represented in the DEGs commonly (com) regulated by flg22 and chitin in *B. rapa*. Gene Ontology (GO) over-representation analysis was carried out using PANTHER classification system (pantherdb.org). Dot size is proportional to the number of DEGs detected for each GO category. Dot color indicates false discovery rate (FDR)

To identify the major biological processes which are regulated by chitin and flg22, we subjected the set of common DEGs to Gene Ontology enrichment analysis (Fig. 4). The DEGs rapidly upregulated are predominantly related to stress response and signal transduction (protein phosphorylation/dephosphorylation, protein ubiquitination, calcium ion transmembrane transport, transmembrane receptor, and G-protein signaling pathways). On the other hand, genes involved in the regulation of growth and auxin-activated signaling were over-represented in the set of common DEGs rapidly downregulated, suggesting that MAMP perception not only sets the defense response in motion but might also lead to a rapid shift in the allocation of resources from growth processes. After 3 h of treatment, the upregulated DEGs were mostly associated with hormones and defense compounds biosynthesis (chorismate, cinnamic acid, sulfur compound biosynthesis, and L-phenylalanine metabolism). Conversely, several processes related to the primary metabolism were over-represented in the DEGs that were commonly downregulated by both MAMPs (starch, chlorophyll, lipid biosynthesis, and light reaction of the photosynthesis), highlighting further the likely shift in resources allocation initiated soon after MAMP perception.

**Fig. 5:**
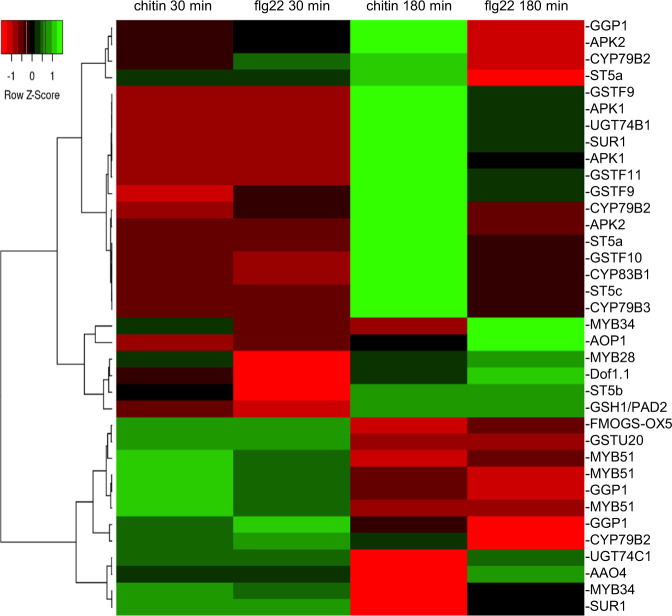
Hierarchical clustering of glucosinolate biosynthesis genes regulated by flg22 and chitin in *B. rapa*. The full list of glucosinolate genes was retrieved from the Brassica database (brassicadb.org/brad/glucoGene.php)

Glucosinolates are Brassicaceae-specific defense secondary metabolites contributing mainly to the response to herbivory but also to innate immunity and in particular to callose deposition at the plant-pathogen contact surface^25,26^. Out of the 102 glucosinolate biosynthesis genes reported in *B. rapa* genome^27^, 36 were differentially expressed in our dataset. Hierarchical clustering of these DEGs revealed that expression of *B. rapa* homologs of *MYB51*, *CYP79B2*, *ST5,* and *SUR1* was rapidly induced by both MAMPs, indicating the activation of the indole-glucosinolate synthesis pathway (Fig. 5). Interestingly, specific copies of glucosinolate synthesis genes were uniquely differentially expressed in the 3 h chitin sample (APK2 *Bra017872*, CYP79B3 *Bra030246*, UGT74B1 *Bra024634*), suggesting that different compounds or their specific accumulation could be regulated by chitin perception (Fig. 5).

Our data also indicated that components involved in response to stress (endochitinase, lignin synthesis, heat-shock proteins) or methylation and transmembrane transport were uniquely up- and downregulated, respectively, after 3 h chitin treatment (Fig. S7a). We confirmed by qRT-PCR in other sets of biological samples that S-adenosylmethionine synthase 3 (*Bra017219*), UDP-glycosyltransferase (*Bra024634*) or endochitinase (*Bra000310*) were uniquely upregulated by the chitin treatment (Fig. S7b), further supporting a specific or at least prolonged transcriptional response elicited by chitin compared to that elicited by flg22.

### Induction of PTI in *B. rapa* effectively restricts pathogen growth

**Fig. 6:**
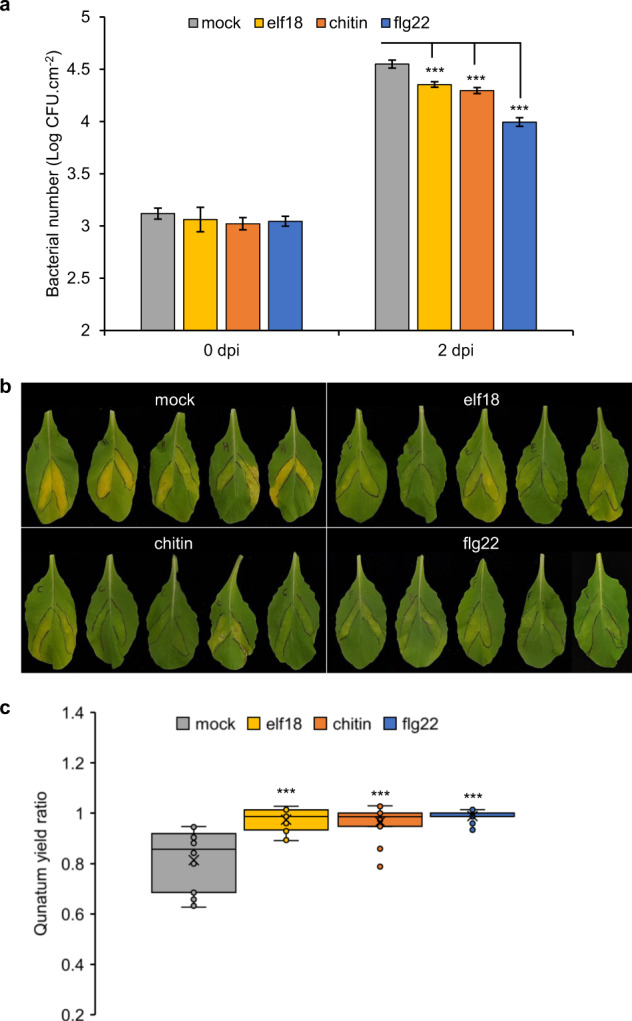
MAMP pre-treatment restricts *Xanthomonas campestris campestris* (*Xcc*) growth in *B. rapa*. Norang seedlings pre-treated for 24 h with water (mock), 100 nM elf18, 100 μg ml^−1^ chitin or 100 nM flg22 were inoculated with *Xcc* suspension. **a** Bacterial enumeration 3 h (0) or 2 days post-inoculation (dpi). Data are log10 of mean number of colony-forming unit (CFU) +/− standard error from three independent biological repeats (*n* = 30). Asterisks indicate statistical significance of difference with mock-treated samples (Student’s *t*-test, *p* < 0.001). **b** Disease symptoms at 6 dpi. Infiltrated areas are circled in black. Photographs are representative of three independent biological repeats. **c** Quantifi**c**ation of disease symptoms at 6 dpi. Data are the quantum yield ratio between inoculated and non-inoculated leaf areas in three independent biological repeats (*n* = 15). Asterisks indicate statistical significance of difference with mock-treated samples (Student’s *t*-test, *p* < 0.001)

Three unrelated MAMPs, elf18, chitin, and flg22 elicited fast physiological and transcriptional responses in *B. rapa*. The final outcome of PTI is an effective restriction of pathogen growth, as demonstrated in Arabidopsis or tomato^4,28^. To determine whether MAMP perception in *B. rapa* could protect plants from infection, we measured the growth of the bacterial pathogen *Xanthomonas campestris campestris* (hereafter *Xcc*), the causal agent of black rot disease in Brassica crops, in leaves pre-treated for 24 h with elf18, chitin or flg22 (Fig. 6a). Although *Xcc* is a vascular pathogen, it can grow significantly in the mesophyll when infiltrated into *B. rapa* leaves^29^. In our conditions, *Xcc* grew over 30-fold within two days in leaves pre-treated with water (mock, Fig. 6a). However, compared to mock pre-treated leaves, leaves pre-treated with MAMPs contained between 2 (elf18, chitin) to 8 (flg22) times less *Xcc* colony-forming units. Furthermore, the chlorosis visible at 6 dpi in mock-treated *Xcc* inoculated leaves was less pronounced in the MAMP-pretreated leaves (Fig. 6b). In an attempt to quantify this chlorosis, we calculated the PSII quantum yield ratio between *Xcc* inoculated and non-inoculated leaf areas. This ratio was close to 1 in the elf18-, chitin- and flg22-pretreated leaves but significantly lower in the mock-pretreated leaves, in accordance with the marked symptoms development (Fig. 6c). These experiments demonstrate that the perception of any of the 3 MAMPs tested could effectively restricts the growth of a virulent pathogen in *B. rapa* leaf.

Altogether these results highlight the robustness of the PTI in *B. rapa*, despite notable differences in the amplitude of physiological and transcriptional changes elicited by different MAMPs.

## Discussion

Syntenic homologs of Arabidopsis characterized PRRs are present in *B. rapa* genome and expressed in vegetative organs. The high degree of conservation is indicative of the critical role of these receptors for *B. rapa* defense against pathogens. Syntenic homolog of RECEPTOR OF EMAX and RECEPTOR-LIKE PROTEIN 30, required for the recognition of the elusive MAMPs from *Xanthomonas* eMAX and from *Sclerotinia* SCLEROTINIA CULTURE FILTRATE ELICITOR 1 respectively, were also detected in *B. rapa* genome although their activity could not be tested, as these ligands are not easily accessible^30,31^. Nonetheless, the rapid production of ROS and the transient up-regulation of *BraNLH10* in Norang seedlings treated with LPS, PGN, elf18, chitin and flg22 indicate that functional PRRs could detect different MAMPs in *B. rapa* leaf.

The Ef-Tu receptor gene *AtEFR* has two syntenic homologs in *B. rapa* genome. *BraEFR1* and *BraEFR2* are both expressed albeit to significantly lower levels compared to *BraFLS2* in vegetative tissues^19^. Both genes encode the conserved receptor domains (21 LRRs, transmembrane, and kinase domains). Both proteins harbor the residues found to be essential for EFR function including D849 essential for EFR kinase activity^32^ and Y836 required for EFR signaling through tyrosine phosphorylation^33^. BraEFR1 and BraEFR2 could accumulate at the cell periphery when transiently expressed in *N. benthamiana*, yet only BraEFR2 conferred gain of elf18 perception in Arabidopsis *fls2 efr cerk1* mutant or in *N. benthamiana* tissue as measured by seedling growth inhibition, ROS production and marker gene expression. Although the cytoplasmic domain of BraEFR2 shows no auto-phosphorylation activity in vitro^34^, we showed that this receptor could function *in planta*. Considering this, we hypothesize that the difference between BraEFR1 and BraEFR2 might reside in their ability to bind ligand. In Arabidopsis, EFR LRR 1 to 5 and 19 to 21 were found to be critical for elf18 binding through an elegant study of chimeric receptors^35^. A similar approach with chimera of BraEFR1 and BraEFR2 could therefore bring new insights into the biochemical requirement of elf18 binding for the receptor activation and clarify whether *BraEFR1* is undergoing pseudogenization or, less likely, is maintained because it confers a new recognition.

The amplitude of ROS production and *BraNHL10* up-regulation triggered by elf18 treatment in *B. rapa* was noticeably lower than that triggered by flg22 treatment despite similarities of the receptor characteristics and function. This difference, also often observed in Arabidopsis, could stem from the more recent acquisition of EFR in the Brassicaceae family compared to FLS2^36^. This could explain EFR higher dependency on glycosylation for proper folding and targeting, or a putative weaker ability to recruit downstream signaling components. Alternatively, the lower amplitude of the physiological responses triggered by EFR activation could be attributed to transcriptional regulation of the receptor accumulation as BraEFR2 is 5 to 50 times less expressed than BraFLS2 or BraLYK5 in the vegetative tissues.

We also observed only modest change in *BraNHL10* gene expression after 30 min to 3 h treatment with LPS or PGN. Since LPS treatment for 24 h and PGN treatment for 6 h regulate the expression of 2139 and 750 genes, respectively in Arabidopsis^17,37^, our data indicate the conservation of the perception pathway for these MAMPs, although longer exposure might be required to measure the full extent of transcriptional changes. Alternatively, we cannot exclude that absence or low amplitude response observed in our study could result from the poor solubility of PGN or the aggregation of LPS into micelles^38^.

The analysis of *B. rapa* transcriptome at early (30 min) and later (3 h) time following chitin and flg22 treatment revealed the massive extent of transcriptional reprogramming elicited by MAMP perception in *B. rapa*, similar to the magnitude of the transcriptional response observed in the closely related species Arabidopsis^4,39^ and *B. napus*^40^. Overlapping and conserved sets of genes involved in stress perception and signal transduction were rapidly upregulated, before the onset of induction of genes involved in defense hormones and antimicrobial compounds synthesis. Conversely, genes commonly downregulated by flg22 and chitin are associated with growth, photosynthesis and primary metabolism regulation. This response is conserved with Arabidopsis and suggests the re-allocation of the plant resources from growth toward the defense responses, in part mediated by repression of auxin-regulated signaling pathways^41,42^.

Intriguingly, we observed stronger, prolonged or specific transcriptional changes in *B. rapa* in response to chitin compared to flg22. This is in stark contrast with Arabidopsis transcriptional responses, as the number of DEGs in response to flg22 treatment in Arabidopsis seedlings, leaves or roots is consistently higher than the number of chitin-responsive genes^39,43^. This is unlikely to be due to our experimental set up, in particular the exposure of the leaf tissue to the flg22 peptide, as we demonstrated that in the same conditions, the flg22 treatment rapidly elicits the production of ROS and efficiently restricts pathogen growth. Hence, our results suggest that chitin perception is differentially regulated in *B. rapa* compared to Arabidopsis and leads to specific responses (e.g., synthesis of different indole-glucosinolate compounds) relevant to anti-fungal defenses. Alternatively, the diverse pathways for negative feedback on the flg22-triggered signaling, such as the ubiquitination and endocytosis of activated FLS2, might rapidly limit the extent of the transcriptional reprograming elicited by flg22 perception in *B. rapa*^44^.

In a recent study, a putative MAMP prepared from yeast cell wall extract was shown to protect *B. rapa* (var *chinensis*) from infection by the bacterial pathogen *Pseudomonas cannabina*^45^. Despite variations in the amplitude of the MAMP-elicited responses or the number and nature of the DEGs observed in our study, we could similarly show that the perception of 3 unrelated MAMPs activated immunity in *B. rapa* and lead to the restriction of pathogen growth. Collectively, these results highlight the pivotal role of PTI for plant defense and further raise the importance of PRRs as target for marker-assisted breeding of disease-resistant vegetable crops.

## Materials and methods

### Plant materials

Norang is a *B. rapa* subsp *pekinensis* commercial cultivar provided by FarmHannong (Korea). Arabidopsis *fls2 efr cerk1* triple mutant is described by Gimenez-Ibanez et al.^21^. Seeds were sown on wet paper towel and kept in high humidity for three days until germinated. Seedlings were then transplanted in soil mixture and grown at 21 °C in short-day conditions (10-h light/14-h dark) in a controlled-environment chamber. *N. benthamiana* were grown at 24 °C in long-day conditions (16-h light/8-h dark) in a controlled-environment chamber.

### Chemicals

The peptides flg22 (TRLSSGLKINSAKDDAAGLQIA) and elf18 (ac-SKEKFERTKPHVNVGTIG) were synthesized by Peptron (Korea). Lipopolysaccharides (from *Pseudomonas aeruginosa* 10), peptidoglycan (from *Bacillus subtilis*) and chitin (from shrimp shell) were purchased from Sigma. All the MAMPs were diluted in ultrapure water and stored at −20 °C before use.

### Molecular constructs

*AtEFR* (At5g20480), *BraEFR1* (Bra006560) and *BraEFR2* (Bra002305) Golden Gate compatible modules were amplified from Arabidopsis Col-0 or *B. rapa* Norang cDNA with flanking *Bsa*I recognition sites at the 5′ and 3′ end (primers listed in Table S4). The sequenced modules were assembled with C-terminal YFP under the control of the CaMV 35S promoter in pICH86988 binary vector (gift from Sylvestre Marillonnet, Addgene #1000000044). The conformity of the assemblies was assessed by restriction analysis, before mobilization into Agrobacterium.

### Agrobacterium-mediated stable transformation of *Arabidopsis thaliana*

*A. thaliana fls2 efr cerk1* plants were transformed using the floral dip method described by Clough and Bent^46^ with *Agrobacterium tumefaciens* AGL1 carrying BraEFR1 or BraEFR2 constructs in pICH86988. Transgenic plants were selected on medium containing 50 μg ml^−1^ kanamycin and two independent homozygous T3 lines with similar level of transgene expression were selected for each construct.

### Agrobacterium-mediated transient expression assays

Agrobacterium-mediated transient transformation of *N. benthamiana* leaf was carried out as described previously^47^. *A. tumefaciens* AGL1 cells were grown on Luria-Bertani (LB) medium with selective antibiotics, centrifuged and resuspended in infiltration medium (10 mM MgCl_2_ and 10 mM MES-KOH, pH = 5.6) to reach OD_600 nm_ = 0.4. The suspensions were infiltrated into fully expanded leaves of 5-week-old *N. benthamiana* plants using a blunt end syringe.

### Seedling growth inhibition

Seedling growth inhibition by elf18 peptide was carried out as described by Schwessinger et al.^32^. Arabidopsis seeds were surface sterilized and allow to germinate on MS medium for 5 days. Seedlings were then transferred into liquid medium containing increasing concentration of elf18 and kept in the controlled-environment chamber (16-h light/8-h dark) for 7 more days before fresh weight determination using a precision scale.

### ROS production

Luminol-based measurement of ROS production was carried out as described by Sang and Macho^48^. Leaf discs (20 mm^2^) of *B. rapa* or Arabidopsis were collected using a biopsy punch and floated on 150 μl of deionized water overnight. The water was replaced with 100 μl of assay solution containing 100 μM luminol (Sigma), 2 μg of horseradish peroxidase, and the tested MAMPs. Luminescence was measured in a relative light unit (RLU) for 75 min using a GloMax 96 microplate luminometer (Promega).

### Subcellular localization of EFR-YFP proteins

*N. benthamiana* leaf tissues were harvested 2 days after Agrobacterium infiltration and observed by confocal microscopy. Images were obtained with a confocal laser scanning microscope (+Super-resolution) SP8X (Leica) using 40× water immersion objective and 514 nm laser for YFP excitation.

### Gene expression by qRT-PCR

Norang or *N. benthamiana* leaf discs (100 mm^2^) were floated overnight on deionized water then treated with different MAMP solutions (50 μg ml^−1^ LPS, 50 μg ml^−1^ PGN, 100 nM elf18, 100 μg ml^−1^ chitin or 100 nM flg22) or water for 30, 60, and 180 min. Total RNA was extracted using Accuzol (Bioneer, Korea) according to the manufacturer’s instructions. RNAs were treated with DNAse I to remove residual genomic DNA and 1 μg total RNA was used for cDNA synthesis with the Maxima first-strand cDNA synthesis kit (Thermofisher). For quantitative RT-PCR, cDNA template was combined with GoTaq qPCR master mix (Promega) and PCRs were performed in triplicate with a LightCycler480 system (Roche) with the primers listed in Table S4.

### RNA-Seq library preparation, sequencing and determination of differentially expressed genes

Three independent biological replicates of 15 Norang leaf discs (100 mm^2^) treated with water, 100 μg ml^−1^ chitin or 100 nM flg22 for 30 and 180 min were generated. Total RNA was extracted using Accuzol (Bioneer, Korea) and treated with DNAse I. RNA samples were quantified and the purity assessed by Agilent chromatography. Libraries of mRNA were constructed from 1 μg total RNA using TruSeq Stranded mRNA LT Sample Prep kit and sequenced using Illumina NovaSeq 6000 sequencing system by Macrogen (Korea). Low quality reads were trimmed using Trimmomatic. The trimmed reads were mapped to *B. rapa* reference genome (Bra chromosomev1.5; brassicadb.org/brad/datasets/pub/BrassicaceaeGenome/Brassica_rapa/V1.0/Bra_Chromosome_V1.5/) using HISAT2. Read count and FPKM were obtained from transcript assembly using StringTie. Differentially expressed genes were called using DESeq2 with log2 fold-change threshold of 2 and nbinomWald Test raw *p*-value < 0.05. Hierarchical clustering of glucosinolate biosynthesis genes was performed using Pearson distance and average linkage in Heatmapper^49^. The transcriptomic data can be found in the Gene Expression Omnibus repository under the accession number GSE150746.

### *Xanthomonas campestris campestris* infection and bacterial growth

The first fully expanded leaves of *B. rapa* Norang 3-week-old seedlings were infiltrated with water, 100 nM elf18, 100 μg ml^−1^ chitin or 100 nM flg22. After 24 h, the same leaves were infiltrated with a 10^5^ CFU ml^−1^ suspension (in 10 mM MgCl_2_) of *Xanthomonas campestris campestris* 8004. Leaf discs (100 mm^2^) were harvested at 3 h and 48 h post-inoculation and ground in 10 mM MgCl_2_. Serial dilutions of the extract were plated on LB medium supplemented with 50 μg ml^−1^ rifampicin and incubated at 28 °C for 48 h before bacterial enumeration. Plants were kept in the controlled-environment chamber (10-h light/14-h dark) up to 6 days after infection to photograph disease symptoms. Quantum yield (Fv/Fm) was determined in a closed FluorCam (Photon System Instruments) following the manufacturer’s instructions.

## Supplementary information

Supplementary information

Supplementary table S3
