# Supplementary material for: Perception of unrelated microbe-associated molecular patterns triggers conserved yet variable physiological and transcriptional changes in Brassica rapa ssp. pekinensis
Source: Hortic Res. 2020 Nov 1;7:186. doi: 10.1038/s41438-020-00410-0 (PMC7603518; doi:10.1038/s41438-020-00410-0)

**Perception of unrelated microbe-associated molecular patterns triggers conserved yet variable physiological and transcriptional changes in *Brassica rapa* spp. *pekinensis*.**

Wanhui Kim^1,2^, Maxim Prokchorchik^3^, Yonghua Tian^1,2^, Seulgi Kim^1^, Hyelim Jeon^1,2^ and Cécile Segonzac^1,2,*^

**Supplementary Information**

**Table S1.** Number of reads mapped to *B. rapa* v1.5 genome assembly in three biological repeats for each condition.

|  |  | **Number of mapped reads** | | | **Percentage of mapped reads** | | |
| --- | --- | --- | --- | --- | --- | --- | --- |
|  |  | **R1** | **R2** | **R3** | **R1** | **R2** | **R3** |
| **WC** | **30 min** | 53,982,615* | 57,470,163 | 56,089,879 | 94.04* | 94.2 | 94.28 |
|  | **180 min** | 56,188,291 | 52,267,748 | 52,984,517 | 93.79 | 94.24 | 94.22 |
| **WF** | **30 min** | 53,982,615 | 63,047,170 | 52,588,082 | 94.04 | 94.09 | 93.82 |
|  | **180 min** | 55,645,909 | 53,989,100 | 47,044,896 | 94.06 | 93.94 | 93.79 |
| **Chitin** | **30 min** | 48,119,138 | 52,023,818 | 63,403,715 | 93.88 | 94.15 | 94.01 |
|  | **180 min** | 49,575,643 | 60,273,570 | 48,280,686 | 94.08 | 94.08 | 93.77 |
| **Flg22** | **30 min** | 46,880,539 | 50,649,498 | 61,026,020 | 93.68 | 93.75 | 93.52 |
|  | **180 min** | 51,076,603 | 57,598,601 | 57,230,578 | 94.1 | 94.08 | 94.07 |

WC: control (water treatment) for chitin

WF: control (water treatment) for flg22

R: Replicate

*: shared with WF 30 min R1

**Table S2.** Correlation analysis between the biological replicates for each condition.

|  |  |  | **R2** | **R3** |
| --- | --- | --- | --- | --- |
| **WC** | **30 min** | R1 | 0.982 | 0.976 |
|  |  | R2 |  | 0.993 |
|  | **180 min** | R1 | 0.976 | 0.973 |
|  |  | R2 |  | 0.991 |
| **WF** | **30 min** | R1 | 0.989 | 0.982 |
|  |  | R2 |  | 0.985 |
|  | **180 min** | R1 | 0.989 | 0.989 |
|  |  | R2 |  | 0.980 |
| **Chitin** | **30 min** | R1 | 0.971 | 0.976 |
|  |  | R2 |  | 0.989 |
|  | **180 min** | R1 | 0.989 | 0.979 |
|  |  | R2 |  | 0.990 |
| **Flg22** | **30 min** | R1 | 0.986 | 0.974 |
|  |  | R2 |  | 0.986 |
|  | **180 min** | R1 | 0.993 | 0.992 |
|  |  | R2 |  | 0.991 |

WC: control (water treatment) for chitin

WF: control (water treatment) for flg22

R: Replicate

Number: Pearson’s coefficient

**Table S3.** List of all *B. rapa* genes differentially expressed in at least one condition. Fold-change (fc) and *p* value (raw.pval) are indicated for each gene (available as excel file). The full data set is also available from Gene Expression Omnibus platform (GSE150746).

**Table S4.** List of primers used in this study.

| **Gene ID** | **Primer name** | **Primer sequence (5' ->3')** |
| --- | --- | --- |
| Bra006560 | BraEFR1_mod1F | GGTCTCAAATGAAGCTCTCTCTTTCACTTGC |
| Bra006560 | BraEFR1_mod1R | GGTCTCAAGGTTTCCTCCAAGGGACAG |
| Bra006560 | BraEFR1_mod2F | GGTCTCAACCTAATCTCTGGAACTCTTCC |
| Bra006560 | BraEFR1_mod2R | GGTCTCATCAGATGGGTTACCGCCACTGG |
| Bra006560 | BraEFR1_mod3F | GGTCTCACTGATTCTTCTACTTTGGGG |
| Bra006560 | BraEFR1_mod3R | GGTCTCACGAATATTGGGTTCTCGATTGAATG |
| Bra002305 | BraEFR2_mod1F | GGTCTCAAATGAAGCCGTTTCTTTCAATTGC |
| Bra002305 | BraEFR2_mod1R | GGTCTCAAGTTCAGTGCAGTTAGTTAAACC |
| Bra002305 | BraEFR2_mod2F | GGTCTCAAACTAGAGTTCTTAGATGCTGG |
| Bra002305 | BraEFR2_mod2R | GGTCTCAAGGTTCAAGACTTTAACAGC |
| Bra002305 | BraEFR2_mod3F | GGTCTCAACCTCTTGAAGCATGGAGCAACG |
| Bra002305 | BraEFR2_mod3R | GGTCTCACGAACATTGTATGCATGTCCGCGC |
| At5g20480 | AtEFR_mod1F | GGTCTCAAATGAAGCTGTCCTTTTCACTTG |
| At5g20480 | AtEFR_mod1R | GGTCTCAAGAGATAAGATTTTGTCCAAGG |
| At5g20480 | AtEFR_mod2F | GGTCTCACTCTGGAACCATTCCTCATGACATCG |
| At5g20480 | AtEFR_mod2R | GGTCTCAAGGTTTCACATTCCGCCATAAAGC |
| At5g20480 | AtEFR_mod3F | GGTCTCAACCTTCAAGGGTATACGACATCG |
| At5g20480 | AtEFR_mod3R | GGTCTCACGAACATAGTATGCATGTCCGTATTTAAC |
| Bra017272 | BraNHL10_qPCR_F | TTCAACGCCGGAGAGTTACG |
| Bra017272 | BraNHL10_qPCR_R | TCCCCAAGCTTAAACCTAACCC |
| Bra006661 | BraEF1alpha 1_qPCR_F | TTGACGGGCGATCTGGAAAG |
| Bra006661 | BraEF1alpha 1_qPCR_R | TCCCTAACAGCGAAACGACC |
| Bra010367 | Bra010367_qPCR_F | ATGGTAATCGTGGCGTTGTT |
| Bra010367 | Bra010367_qPCR_R | CCAAACGCTGAAACCAAAA |
| Bra009738 | Bra009738_qPCR_F | TCTACGCAAGCCAATGTCAG |
| Bra009738 | Bra009738_qPCR_R | CTAGGGTTCATGCCACCAAT |
| Bra020747 | Bra020747_qPCR_F | TCAATCCCAAAAGGTTGGAG |
| Bra020747 | Bra020747_qPCR_R | TCCACCCCCAAAGAGTAAGA |
| Bra031321 | Bra031321_qPCR_F | TCCCCGAGCTACGTAAGAGA |
| Bra031321 | Bra031321_qPCR_R | TAGCTTTGGTCTGCGTGTTG |
| Bra017219 | Bra017219_qPCR_F | ACTCACCGGGAGGAAGATCA |
| Bra017219 | Bra017219_qPCR_R | ACGACACCTGGACAATGCAG |
| Bra024634 | Bra024634_qPCR_F | CCAGAAGGGTTTGTGGAAGC |
| Bra024634 | Bra024634_qPCR_R | CGGAACTCCCAAACTCAACC |
| Bra000310 | Bra000310_qPCR_F | GCGTTCTTTGCTCACGTCAC |
| Bra000310 | Bra000310_qPCR_R | GTTCCAGGAGAGCTGGATCG |

**Fig. S1.** Comparison of genomic regions containing PRR genes in Arabidopsis and *B. rapa*. Syntenic homologs of Arabidopsis PRRs were identified using the syntenic gene tool search from the Brassica database (brassicadb.org/brad/searchSyntenytPCK.php). Synteny between the genome positions of the identified genes is visualized with the GEvo tool of CoGe (genomevolution.org/coge/GEvo.pl). The *B. rapa* homologous genes are depicted in yellow (**a** CERK1, **b** LYK5, **c** LYM2, **d** EFR, **e** FLS2, **f** LYM1, **g** LYM3, **h** LORE). Colored wedges and lines indicate the collinearity with the corresponding genomic region (40 kb) in Arabidopsis.


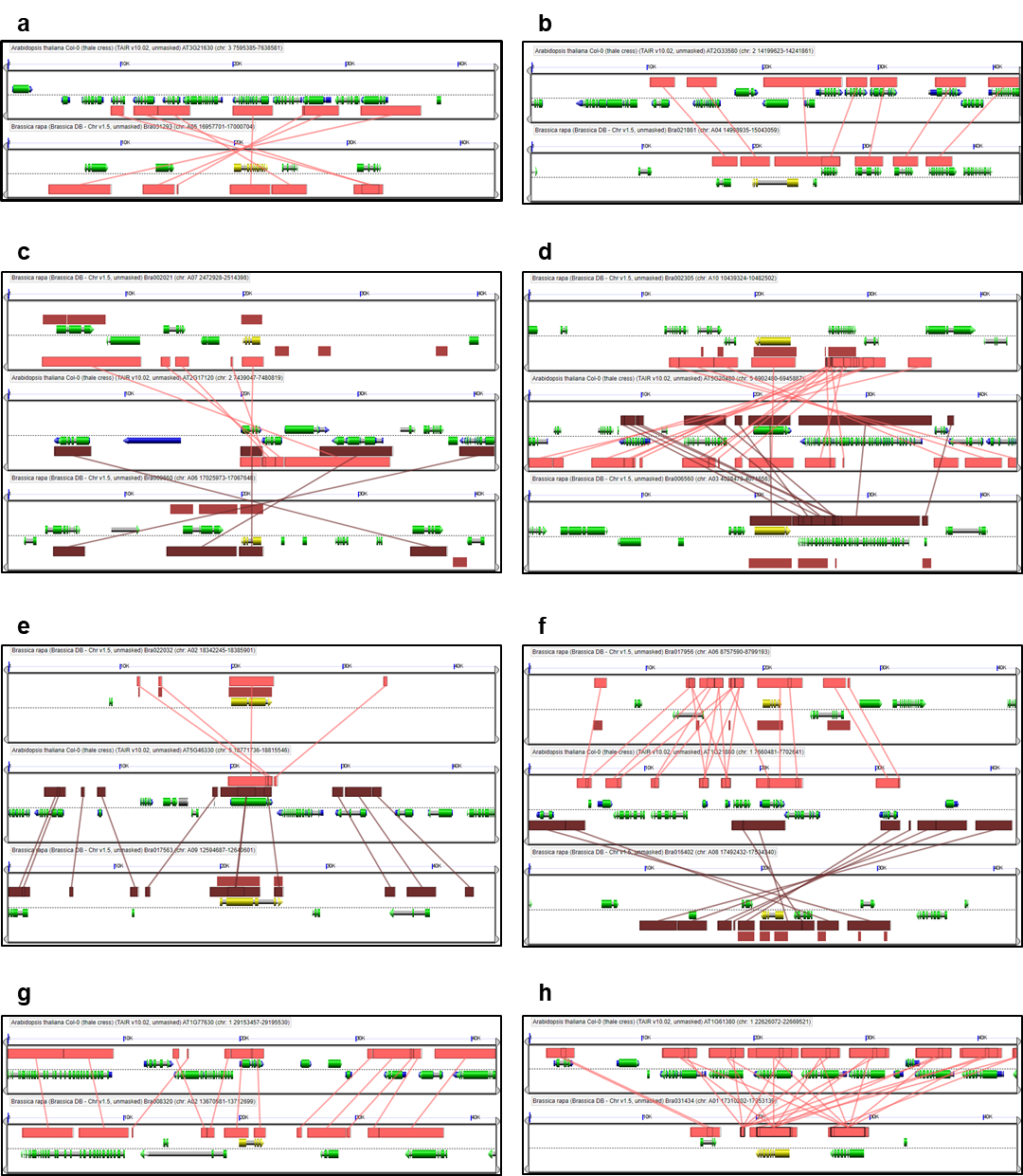


**Fig. S2.** Comparison of *A. thaliana* and *B. rapa* EFR protein sequences. **a** Protein sequence alignment was performed with Geneious. Predicted domains are annotated on AtEFR sequence (pink: signal peptide; yellow: N- and C-terminal regions; orange: Leucine-rich repeats (LRR); red: transmembrane domain; blue: protein kinase domain; brown: ATP-binding site; dark blue: proton-acceptor site). **b** Pairwise distance between AtEFR, BraEFR1 and BraEFR2 is shown as percentage of amino acid identity.


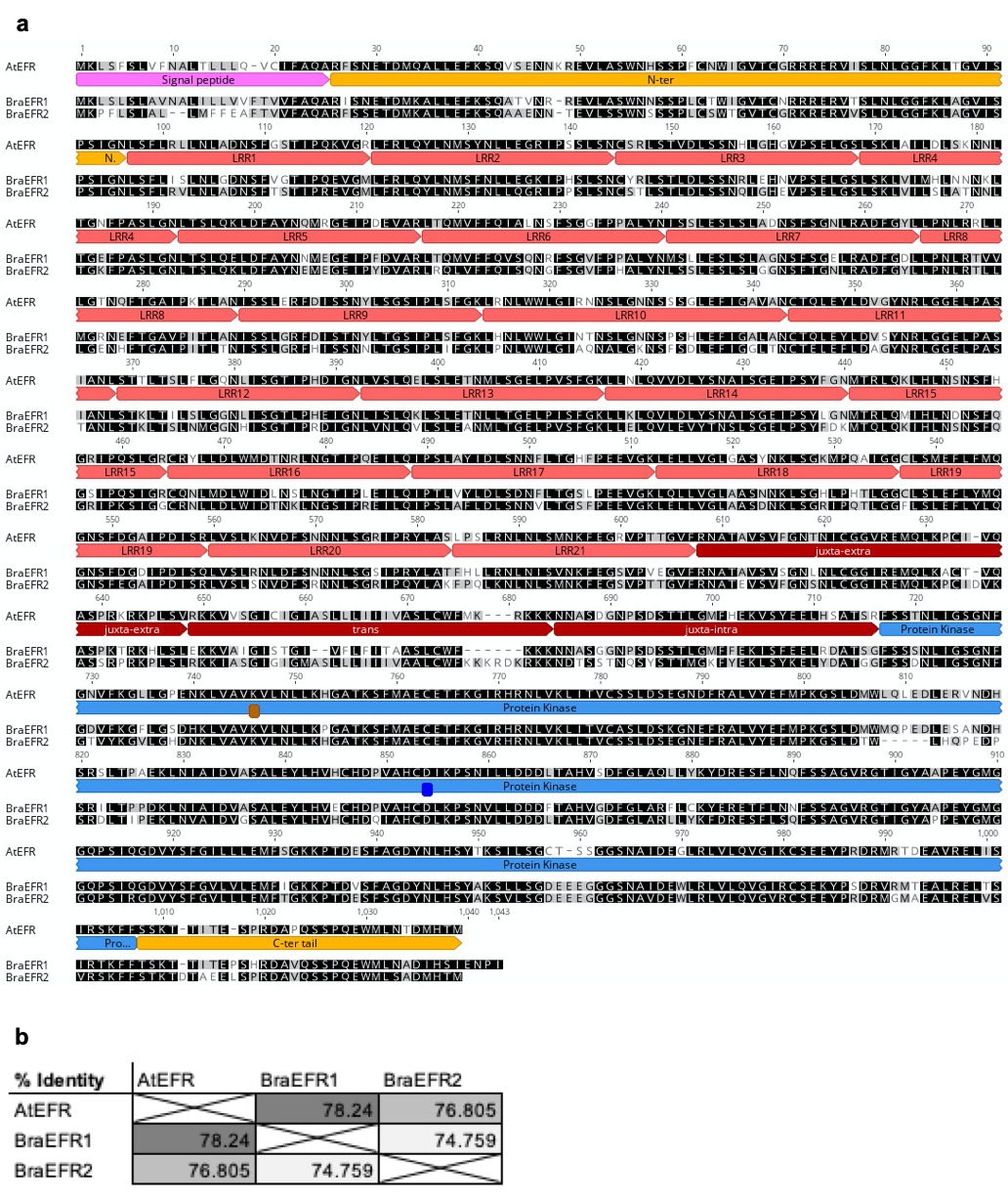


**Fig. S3.** Molecular characterization of Arabidopsis transgenic lines carrying *BraEFR* alleles. **a** Schematic of EFR cDNA sequence in *A. thaliana* and *B. rapa*. EFR-CAPS forward (f) and reverse (r) primers are identical in AtEFR, BraEFR1 and BraEFR2. Specific restriction sites (position) and size of the EFR-CAPS amplicon are indicated for each allele. **b** *EFR* expression in *A. thaliana* wild type (Col-0), *fls2 efr cerk1* (*fec*) mutant and two independent *fec* transgenic lines carrying either *BraEFR1* (*fec*/BraEFR1-1, *fec*/BraEFR1-2) or *BraEFR2* (*fec*/BraEFR2-1, *fec*/BraEFR2-2). Top panel shows EFR-CAPS amplification, middle panel shows the same amplicons digested with *Eco*RI and *Pst*I, bottom panel shows *EF1α* amplicon to attest equal amount of cDNA.


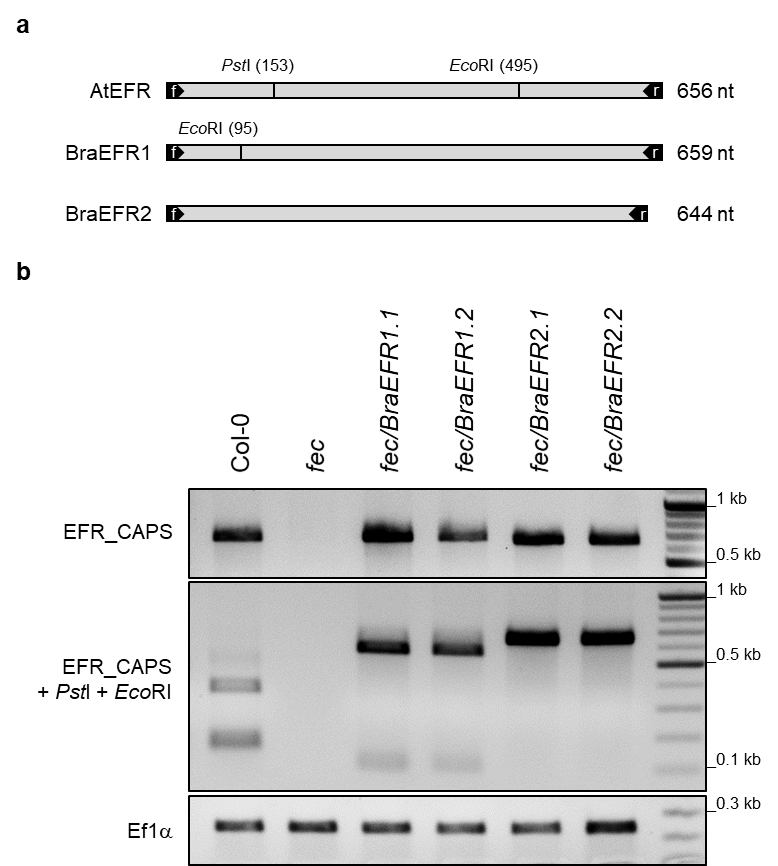


**Fig. S4.** BraEFR-YFP transient expression in *N. benthamiana*. Leaves expressing GFP, AtEFR, BraEFR1 or BraEFR2 were treated with water (mock), 100 nM elf18 or 100 nM flg22. **a** *NbCYP71D20* expression normalized by *NbEF1α* and relative to mock-treated GFP sample. Data are mean values +/- standard error from three technical replicates of one representative out of three biological repeats. **b** BraEFR-YFP subcellular localization was observed by confocal microscopy. Scale bar represents 40 μm.


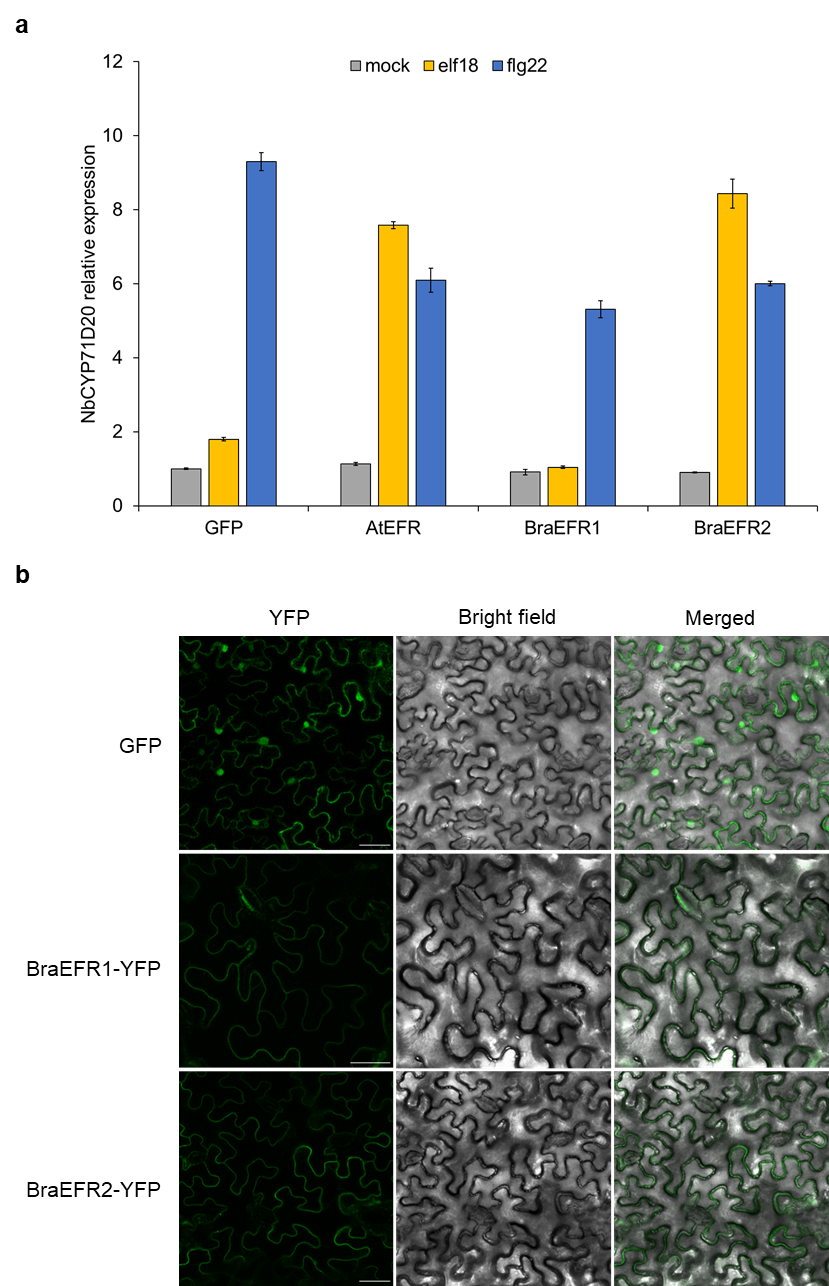


**Fig. S5.** Kinetic of MAMP-elicited ROS production in *B. rapa*. Leaf discs were treated with water (mock), or increasing concentration of elf18 (**a**), chitin (**b**) or flg22 (**c**) for 75 min. Data are mean values +/- standard error of relative light unit (RLU) from three independent biological repeats (n=48).


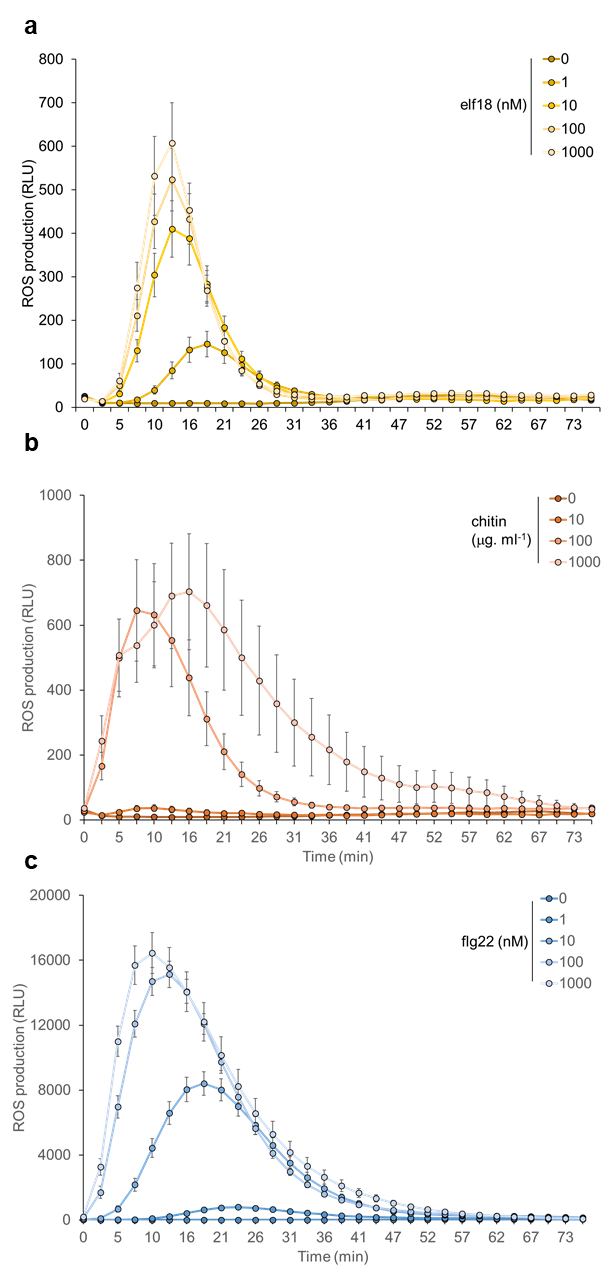


**Fig. S6.** Expression analysis by quantitative PCR of selected DEGs identified from the RNA-Seq analysis. *Bra010367*, *Bra009738*, *Bra020747* and *Bra031321* expression normalized by *BraEF1α* (qPCR, blue bars) compared to expression determined by RNA-Seq analysis (FPKM, green circles). Data are mean values +/- standard error from three independent biological repeats for each method.


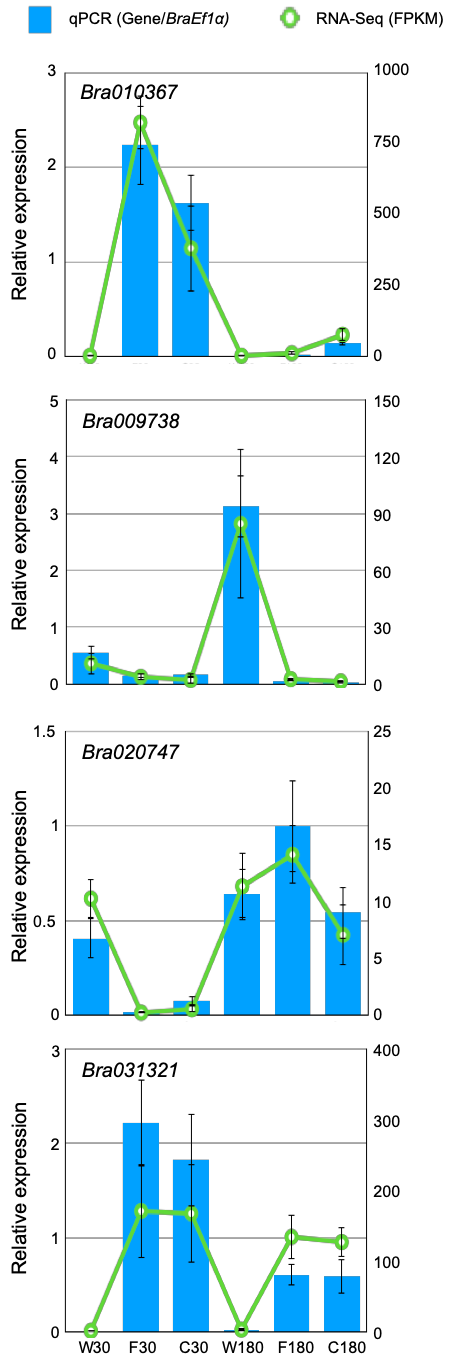


**Fig. S7.** Biological functions over-represented in the DEGs uniquely regulated by chitin in *B. rapa*. **a** Gene Ontology (GO) over-representation analysis was carried out using PANTHER classification system (pantherdb.org). Dot size is proportional to the number of DEGs detected for each GO category. Dot color indicates false discovery rate (FDR). **b** Expression analysis by quantitative PCR of the genes identified as uniquely regulated by chitin after 3 h of treatment. *Bra017219*, *Bra025634* and *Bra000310* expression normalized by *BraEF1α* (qPCR, blue bars) compared to expression determined by RNA-Seq analysis (FPKM, green circles). Data are mean values +/- standard error from three independent biological repeats for each method.


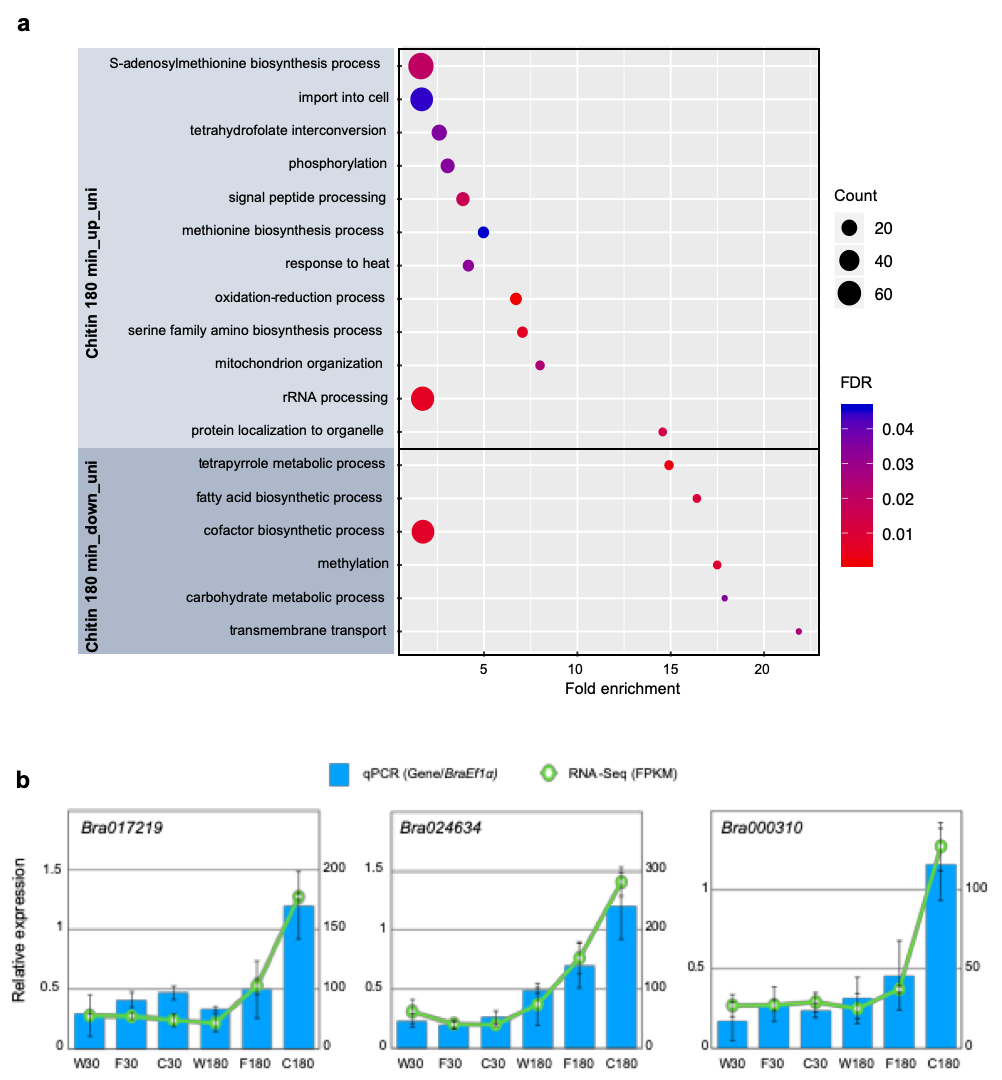

Supplement: Supplementary file 1 — Supplementary information [file 41438_2020_410_MOESM1_ESM.docx]
